# 1431. Dosing Matters – Consistency Analysis of Touch-Free Automatic Hand Hygiene Dispensers

**DOI:** 10.1093/ofid/ofad500.1268

**Published:** 2023-11-27

**Authors:** James W Arbogast, Nicole M Smith, Szava Bansaghi, Nanshan Chen, Travis B Neal, John J McNulty, Tamas P Haidegger

**Affiliations:** GOJO Industries, Inc., Akron, Ohio; GOJO Industries, Inc., Akron, Ohio; Obuda University, Budapest, Budapest, Hungary; The Ohio State University, Columbus, Ohio; GOJO Industries, Inc., Akron, Ohio; GOJO Industries, Inc., Akron, Ohio; Obuda University, Budapest, Budapest, Hungary

## Abstract

**Background:**

Touch-free automatic alcohol-based hand rub (ABHR) dispensers are used extensively in healthcare settings to facilitate hand hygiene (HH). U.S. Centers for Disease Control and Prevention and the World Health Organization HH guidance suggests ABHR dosing per use should be enough to cover the whole hand and keep hands wet for at least 15-20 seconds. Leapfrog 2022 guidance states each activation needs to produce at least 1.0 ml liquid volume. The objective of this study was to determine mean dose volumes and dispensing consistency for five leading US foam ABHR dispenser systems and to assess design impact on dosing variability.

**Methods:**

In total, over 15 dispensers and 32 distinct refills were tested, with >10,000 dispenses analyzed for each of the five systems. Automated testing used computer programming and custom mechatronics to activate the dispensers and capture weights at predetermined delay patterns. Low, medium and high usage patterns of 7x, 48x and 100x activations per day were set with dispense delay patterns based on cluster analysis of actual ABHR dispenser usage patterns from time stamped HH monitoring. In another laboratory, measurements were carried out manually; 50 doses were rapidly collected and weighed within an hour for each refill. Density values were used to calculate dispensed volumes. A linear mixed effects model was fit, and total variance components were compared using a 2-Sample Variance Test.

**Results:**

Three of the dispenser systems had mean ABHR output >1.0ml, and two were < 1.0ml (see Table 1). Test results are compiled into histograms (Figures 1 & 2) to visually display the large differences in average dose and consistency between doses. Dispensers #A & #B have significantly greater variability (p < 0.001), which is driven by pump design used to foam the ABHR.

**Figure:**
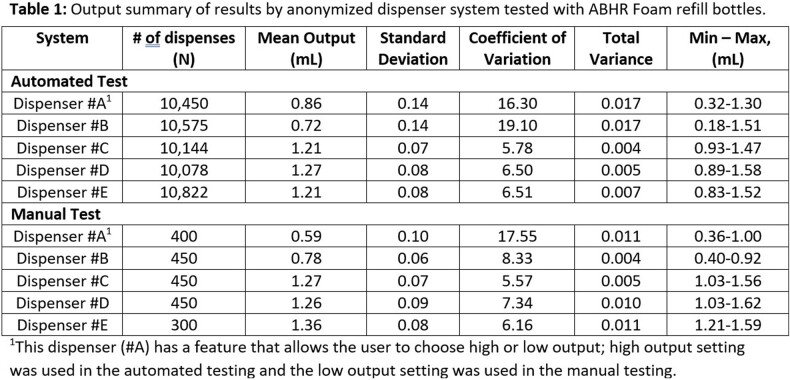

**Figure:**
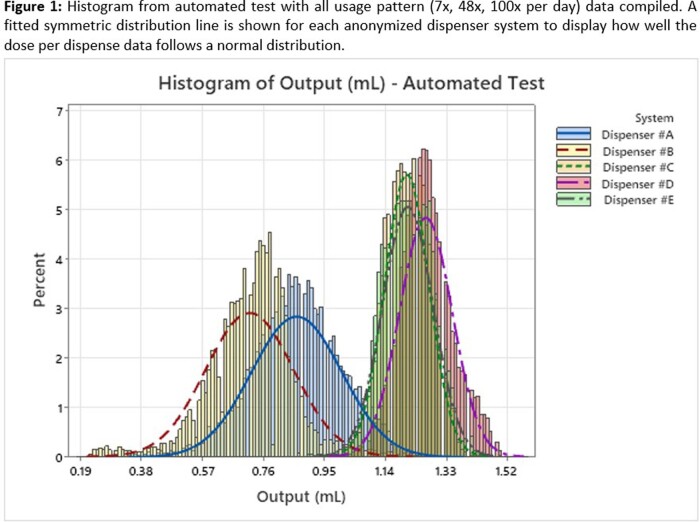

**Figure:**
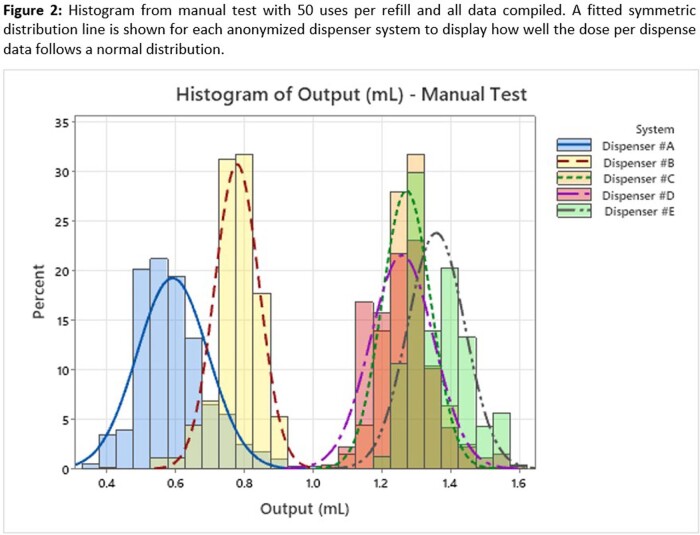

**Conclusion:**

Testing patterns (timing delays between dispenses) impacts dosing performance. The dispenser design and engineering cause significant differences in volume dispensed and consistency across dispenses. Using enough ABHR to cover hands completely and keeping hands wet long enough to significantly reduce pathogens is an important requirement. Facilities should assess ABHR dispenser outputs and consider consistent dosing as an essential performance criterion for effective HH policies and practices.

**Disclosures:**

**James W. Arbogast, PhD**, GOJO Industries, Inc.: employee **Nicole M. Smith, n/a**, GOJO Industries, Inc.: employee **Szava Bansaghi, n/a**, GOJO Industries, Inc.: Grant/Research Support **Nanshan Chen, n/a**, GOJO Industries, Inc.: Grant/Research Support **Travis B. Neal, n/a**, GOJO Industries, Inc.: employee **John J. McNulty, n/a**, GOJO Industries, Inc.: employee **Tamas P. Haidegger, PhD**, GOJO Industries, Inc.: Grant/Research Support

